# Editorial: Molecular Role of Lipids in Aging

**DOI:** 10.3389/fragi.2022.946884

**Published:** 2022-06-20

**Authors:** Dorota Skowronska-Krawczyk, Priyanka Narayan, Peter Tessarz

**Affiliations:** ^1^ Department of Physiology and Biophysics, Department of Ophthalmology, Center for Translational Vision Research, School of Medicine, University of California Irvine, Irvine, CA, United States; ^2^ Genetics and Biochemistry Branch, National Institute of Diabetes and Digestive and Kidney Diseases and National Institute of Neurological Disorders and Stroke, National Institutes of Health, Bethesda, MD, United States; ^3^ Center for Alzheimer's and Related Dementias, National Institutes of Health, Bethesda, MD, United States; ^4^ Max Planck Institute for Biology of Ageing, Cologne, Germany

**Keywords:** lipids, aging, metabolism, membranes, neurodegeneration, epigenetics

Lipids are one of the most understudied groups of biomolecules in the body. Despite their high abundance and important role as a building blocks, metabolites, signalling intermediates, and energy sources, their molecular role is still poorly understood. Different types of lipids (fatty acids, glycerides, nonglyceride, and complex lipids) are supported by a plethora of enzymes, receptors, transporters, and other functional proteins. Destabilization of these processes has important consequences for cell and organismal health. There is a long history of work seeking to identify correlations between the composition of different lipid components and the progression of aging across many tissues. The main hindrance in understanding these processes was the lack of adequate methods of detection and analysis. With the recent progress in analytic and high throughput technologies, we have begun to understand the contribution of lipids to human health. Genome-wide association studies have found several lipid-related genomic variants to be associated with age-related diseases, provoking molecular studies aimed to decipher the extent of the biological role of lipids in normal and pathological aging. Also, aging is associated with an increased risk of lipid-related disorders. Altogether, these findings have propelled interest in lipid biology and the changes in metabolism of lipids in aging. There is now a concerted effort to understand better the molecular role of lipids in health and disease. These studies have spanned a variety model organisms and systems. For example, lipid supplementation studies in multiple model organisms have revealed important functions of lipids in aging including extension of healthspan. Pioneering studies on non-vertebrates (e.g., *C. elegans* and *D. melanogaster*) stimulated the development of the field and encouraged the use of rodents and other animals in understanding the role of lipids in aging. The field is constantly growing, and thanks to the concerted efforts of labs all over the world, we begin to understand the association between lipids, aging, and age-associated diseases.

Lipid biology is intricately linked to several different hallmarks of the aging process. Potent aging interventions, such as dietary interventions have been shown to modulate lipid composition. The next years will see an increase in research that aims at understanding the connection between lipids and individual age-related pathways. One such connection that warrants further investigation, is the link between lipid biosynthetic pathways, lipid composition and epigenetic mechanisms. The epigenome is an important contributor to the regulation of gene expression and the maintenance of cellular states and has been implicated as a major contributor to aging. Over recent years it has become apparent that the writing and removal of epigenetic marks is intricately linked with metabolic processes (Etchegaray and Mostoslavsky, 2016). This process is well understood in the case of histone acylation, particularly in the case of acetyl-CoA. Here, acetyl-CoA as a central metabolite serves as a donor for the modification of lysine residues within histones. Acetyl-CoA can be derived either from acetate (a preferred pathway in *Sacharomyces cerevisiae* (Takahashi et al., 2006)), glucose (Wellen et al., 2009) or via the breakdown of fatty acids (McDonnell et al., 2016). These processes directly couple the nutritional status of cells to chromatin states and gene regulation. Importantly, excess of lipid-derived acetyl-CoA can trigger different transcriptional programs than glucose-derived acetyl-CoA (McDonnell et al., 2016). However, how this specificity is achieved remains unclear, but might be linked to specific transcription factors that mediate the gene expression program. Next to acetyl-CoA, short-chain fatty acids, such as propionyl-, butyryl- or crotonyl-CoA can also be used by histone acetyl transferases are to modify histone lysine sidechains (Sabari et al., 2017), further expanding the epigenetic modification landscape and adjusting the cellular response to environmental conditions. Furthermore, lipids and chromatin modifications share another common precursor, S-adenosyl methionine (SAM) (Ye et al., 2017). SAM is the universal methyl donor for all cellular methylation reactions, such as lipid, histone and DNA methylations. In yeast, loss of phospholipid methylation leads to an increase in histone methylation on several lysine side chains, likely because more SAM is available for histone methylation (Ye et al., 2017). Thus, lipid metabolism might directly regulate histone marks by changing metabolite availability and thus influence gene expression programs. In summary, we now know that lipid metabolic pathways directly impinge on chromatin and gene regulation. How this interplay is connected to complex physiological processes, such as neurodegeneration and/or aging is still largely unexplored, but certainly an exciting research area for the years to come.

Next to the more indirect influence on chromatin via post-translational modifications, lipids have been implicated in a more direct role on chromatin architecture. Several studies have identified lipids to be constituent of chromatin, including phospholipids, cholesterol and phosphatidylcholines (Fernandes et al., 2018). Recently, biophysical studies showed that cholesterol can assist in chromatin folding *in vitro* (Silva et al., 2017). A direct impact of lipids on chromatin architecture and hence, on gene regulation would be an additional route by which metabolic states and epigenome would be coupled. Such a link would certainly be important to understand in the case of aging and/or pathophysiological conditions, in which lipid compositions were altered. However, as the role of lipid-chromatin interaction is currently restricted to observations, more mechanistic studies are required to establish a direct role of lipids on chromatin states.

More traditionally, lipids are perceived as main component of the cell membrane and subcellular structures. Not surprisingly, alterations in membrane lipid composition influence membrane structure and properties. Age-dependent changes in lipid compositions have long been observed, both in total lipid abundance and region-dependent lipid composition but little is known about the mechanism of these changes as well what are the exact consequences of lipid-induced changes in membrane structure in aging-related phenotypes. Lipid composition has been long correlated with membrane fluidity, therefore changes of lipid content, length of chain or saturation of side chains that happen with aging are predicted to affect the homeostasis of the lipid bilayer. For example, “rigid” cholesterol, sphingolipids, and saturated FAs acyl chain were noticed to be accumulated in aging brain (Cutler et al., 2004), liver (Seo et al., 2019) and eyes (Deeley et al., 2010), while flexible, polyunsaturated fatty acids (PUFAs) have generally been observed to drop in CNS during aging (Joffre et al., 2020). Manipulation of membrane phospholipids and PUFAs by n-3 PUFA or n-6 PUFA rich diet showed augmented intramitochondrial Ca^2+^-dependent process with low cardiac mito membrane n-3/n-6 PUFA ratio (Pepe et al., 1999). In the CNS, changes in lipid composition also influence the intercellular signaling and the survival of nerve cells. For example, VLC-SFAs, which are incorporated in sphingolipids that are enriched in synaptic vesicles and regulate synaptic release kinetics and epileptogenesis (Hopiavuori et al., 2018). VLC-PUFAs are critical to photoreceptor survival (Bennett et al., 2014). Finally, the oxidation of PUFAs in aging also alters membrane structure and function (De La Paz and Robert, 1992). Altogether, age-related changes in lipids have profound impact on cellular homeostasis.

Many late-onset neurodegenerative diseases like Alzheimer’s disease (AD), Dementia with Lewy Bodies (DLB), Frontotemporal Dementia (FTD) and Parkinson’s disease (PD) have traditionally been viewed as proteinopathies since the presence of protein aggregates is a primary pathological hallmark (Soto and Lisbell, 2008). However, recent genetic and functional studies have revealed that the disruption of lipid homeostasis can underlie risk for sporadic forms of these diseases (Yadav and Neeraj, 2014; Fanning et al., 2020; Farmer et al., 2020). Over the past decade, genome wide association studies exploring risk for AD, DLB, and PD as well as other neurodegenerative diseases, have all implicated polymorphisms in genes that encode proteins involved in lipid binding and metabolism (Geiger et al., 2016; Nalls et al., 2019; Bellenguez et al., 2022). For example, these include secreted lipoproteins like Apolipoprotein E (*APOE*), Clusterin (or Apolipoprotein J, *CLU*), lipid transporters like LRP1, enzymes that act on membrane lipids like Inositol Polyphosphate-5-Phosphatase D and F (*INPP5D* and *INPP5F*), and lysosomal enzymes like glucocerebrosidase (*GBA*) and granulin (*GRN*). In fact, disrupted lipid accumulation in glia was one of the first hallmarks identified by Alois Alzheimer in his seminal 1907 case report that first described AD (Alzheimer et al., 1995). Now with the advent of more lipidomics, the centrality of lipid disruptions in these diseases has reemerged.

Increased access to lipidomics has enabled large scale studies of the lipidome in several model systems from cells to animal models to human post-mortem samples. These omics studies coupled with functional characterization have revealed the broad cellular and tissue-level consequences of alterations in key lipid risk genes like *APOE* and *TREM2* (Nugent et al., 2020). Multiple studies have revealed that *APOE4* alter cellular lipid state, and that alterations to lipid state can have changes to cell autonomous or non-autonomous functions (Lin et al., 2018; Farmer et al., 2019; Ioannou et al., 2019; Blanchard et al., 2020; Sienski et al., 2021; Victor et al., 2022). These observations have been able to transcend disparate model systems from mice to stem-cell derived brain tissue, to post-mortem human samples (Farmer et al., 2021; Novotny et al., 2021). With the increasing number of risk factors still being identified for many of these diseases (Bellenguez et al., 2022), we expect that similar studies in the future will be able to enumerate the effects of new polymorphisms on cellular and organismal lipid state.

Despite the recent boom in interest in lipid biology in neurodegeneration, multiple opportunities for further growth remain. Since lipidomics analyses often require large sample quantities compared to proteomics and transcriptomics, the development of detection methods that can achieve similar depth of coverage with smaller samples is crucial. New methods like single-cell metabolomics and spatial metabolomics are now allowing for greater resolution of metabolite signals from complex environments (Alexandrov 2020; Seydel 2021). Expansion of use and further development of these techniques will greatly enable the study of precious and complex samples. In addition, the ability to faithfully distinguish signals from similar metabolite species is often performed or checked manually; new computational tools to better deconvolve signals will greatly enhance current lipidomics workflows. The comparison of a growing number of datasets performed with different standards and protocols and in different organisms will require further development of computational tools. In addition, the integration of lipidomics data with proteomics and transcriptomics through modeling will allow for further understanding of the underlying biology driving observed lipid changes.

The fast-growing field of understanding lipids and neurodegeneration shows much promise for future investigation and innovation. Many groups have studied the cellular and organismal consequences of lipid-related neurodegeneration risk factors for years and identified a panoply of seemingly unrelated phenotypes (Liu et al., 2013). Given the involvement of lipids in nearly every cellular process, it is attractive to speculate that these disparate phenotypes may all originate from upstream disruptions to cellular lipid homeostasis. If we can identify and target these upstream disruptions, we may be able to address devastating neurodegenerative diseases therapeutically or preventatively.

In this Research Topic entitled “*Molecular Role of Lipids in Aging*”, several studies and reviews explore the consequences of aging on lipid composition or alterations in lipid metabolic pathways that are connected to either normal or pathological aging.

Gille et al. review the current literature with respect to age-related alterations in several lipid classes focusing on the aging brain and the cardiovascular system. They then move on to discuss the impact of dietary interventions, such as caloric restriction or intermittent fasting and the corresponding reduction in the levels of triacylglycerol, total cholesterol and low-density lipoprotein cholesterol. Importantly, these lipid classes have been associated with age-related diseases when levels increase. However, fatty acids are important for a balanced diet and thus, also supplementation of lipids to diets can have positive effects on the aging process. The authors conclude that lipids provide a link between homeostasis and age-related phenotypes and targeting the lipidome, particularly in a personalized fashion, might be a potent way to increase life- and health-span.

In another original research article, Hänschke et al. addressed the functionality of Lipase 3, a *Drosophila* homolog of the human LIPA gene, which hydrolyzes cholesteryl ester and triacylglycerols. In contrast to human LIPA, Lipase 3 turned out to be a putative phospholipase as knock-outs led to the accumulation of phosphatidylinositol. While expressed at fairly low levels under normal physiological conditions, Lipase 3 is strongly upregulated in starved larvae and female flies as well as in aged males. While the mechanism of Lipase 3 upregulation is not clear, the data indicate that Lipase 3 is involved in the response upon prolonged nutrient deprivation and/or aging.

The last years have seen the development of age predictors based on omics data, also commonly referred to as “aging clocks”. As part of this Research Topic, the group of Unfried et al. added a LipidClock to this portfolio. Based on lipid composition as measured by lipidomics, LipidClock predicts the biological age of wildtype nematodes with a mean absolute error of 1.45 days and is able to simulate survival curves of known long- and short-lived *C. elegans* strains. This proof-of-concept study paves the way for the future development of similar predictors in mammals with the potential to provide further insight into lipid related mechanisms of aging.

A review by Li and Kim centers around a diverse class of bioactive lipids, sphingolipids. The dysregulation of sphingolipids has most commonly been associated with a class of rare, deadly, monogenic diseases occurring early in life. However, advances in technology to detect, quantify, and manipulate sphingolipids have helped illuminate their key role in aging and common age-related diseases. Sphingolipids can modulate central cellular pathways associated with aging including nutrient sensing, cellular senescence, and protein homeostasis. Many of the same pathways that sphingolipids modulate in normal aging become further dysregulated in age-associated diseases like Alzheimer’s disease, Parkinson’s disease, and many cancers. Emerging therapeutics modulating sphingolipid pathways or using sphingolipids as biomarkers have the potential to address the adverse effects of aging and age-associated diseases.

One of the challenges in the field is to know the exact spatial tissue and subcellular localization of different lipid species. Li et al. present the how one can use the D2O probing and stimulated Raman scattering (DO-SRS) microscopy to image the *de novo* lipogenesis in young and old ovaries. The resolution of the method allows to visualize subcellular localization of different classes of lipids, an important achievement in the field of lipidomics.

All the analysis described above would not be possible without the sophisticated technologies. In the short review by Guo et al. authors describe recent developments in the field, pointing out the most recent dynamic improvements and novel approaches in studying the lipid composition, lipids quantification and exact lipid localization. But there is still a lot to be done on the technological level if we want to understand the role of this complex biomolecules in aging. Authors discuss limitations of current methods and give couple of potential ideas for future developments.

The studies published in this Research Topic highlight the important role of lipids in the process of aging and the development of age-related diseases. Given the recent technological developments in lipid analysis, we are convinced that the next decade will see a flurry of activity in this exciting field and we are very much looking forward to these discoveries.
